# School-based eating disorder prevention programmes and their impact on adolescent mental health: systematic review

**DOI:** 10.1192/bjo.2024.795

**Published:** 2024-11-06

**Authors:** Rosa S. Wong, Bianca N. K. Chan, Sok Ian Lai, Keith T. S. Tung

**Affiliations:** Department of Special Education and Counselling, The Education University of Hong Kong, Hong Kong; Department of Paediatrics and Adolescent Medicine, The University of Hong Kong, Hong Kong

**Keywords:** Eating disorder prevention programmes, adolescent, mental health, body acceptance, dissonance-based approach

## Abstract

**Background:**

Growing evidence indicates an association between disordered eating and a range of mental health problems, including anxiety, depression and emotional dysregulation.

**Aims:**

This study aimed to explore whether reducing risk factors for eating disorders, such as body dissatisfaction and low self-esteem, through school-based programmes can enhance adolescent mental health.

**Method:**

We searched PubMed, PsycINFO, EMBASE and Web of Science from the date of inception to 15 October 2023. Data were synthesised by using a systematic narrative synthesis framework, and formal assessments were conducted to assess the quality of the included studies.

**Results:**

After title/abstract screening and full-text assessment, 13 articles met the pre-specified inclusion criteria, comprising a total of 14 studies (*n* = 5853). Notably, three studies encompassed multiple programmes, leading to the identification of 17 eating disorder prevention programmes. Among these programmes, seven (41%) employed dissonance-based approaches. Topics covered in the programmes included psychoeducation, body acceptance, sociocultural issues, nutrition and physical activities, self-esteem and stress coping. Ten (59%) of the programmes were effective in improving adolescent mental health. Six of the 14 studies (43%) did not specify follow-up time, and quality assessments found the majority to be of either high (five studies, 36%) or fair (eight studies, 57%) quality.

**Conclusions:**

The findings from the ten effective programmes consistently support the use of body acceptance strategies in improving the mental health of adolescent students. Brief interventions delivered by trained, non-licensed facilitators appear good for the sustainable implementation of in-school psychological services to support well-being among adolescents.

Eating disorders encompass abnormal eating patterns that may or may not lead to substantial fluctuations in body weight, alongside disturbances in attitudes and behaviours concerning food and body weight.^1,2^ Eating disorders typically begin during adolescence, with the highest rates occurring between the ages of 15 and 19 years.^3^ It is also common for these disorders to continue into adulthood.^4^ According to the diagnostic and statistical manual of mental disorders, eating disorders have three main types, namely anorexia nervosa, bulimia nervosa and binge eating disorder (BED).^5^ The pooled lifetime and 12-month prevalence rates for eating disorders were found to be 0.91 and 0.43%, respectively, with specific lifetime prevalence rates of 0.16% for anorexia nervosa, 0.63% for bulimia nervosa and 1.53% for BED.^6^ Furthermore, eating disorders were associated with increased social and economic costs.^7^ In 2019, eating disorders accounted for 6.6 million of disability-adjusted life-years (DALYs), which is equivalent to 0.3% of the global DALYs.^8^ In addition to reduced workforce and productivity, the loss of well-being caused by eating disorders can result in increased healthcare costs.^7^

Recent research has indicated that emotional dysregulation is an important transdiagnostic risk factor across different types of eating disorder.^9,10^ Eating disorders are often comorbid with emotional problems such as depression and anxiety.^11^ It has been reported that 19.5% of patients with eating disorders experience major depression, and 48.7% show clinically significant depressive symptoms.^12^ Individuals with eating disorders face similar challenges to those with psychiatric disorders, including inadequate coping mechanisms and struggles in handling negative emotions.^13,14^ They tend to adopt maladaptive eating behaviour as a means of regulating their emotions and reducing their psychological distress.^13,15,16^ For instance, people with BED and bulimia nervosa use binge eating to cope with negative emotions,^16,17^ whereas people with anorexia nervosa restrict their food intake or purge to maintain their desired body shape and boost their confidence.^18^

In recent years, more work has been done to develop eating disorder prevention programmes during adolescence, which is a period of heightened vulnerability to both eating and emotional problems.^19^ Meta-analytic reviews have shown that eating disorder prevention programmes that focus on modifying lifestyle, using dissonance-based approaches or enhancing self-esteem have been effective in reducing the risk of developing eating disorders in the future.^20,21^ However, not all interventions have been evaluated for their effects on negative emotions. Even among those that have been studied, the findings have been inconsistent. For instance, a combined dissonance and mindfulness-based eating disorder prevention programme was found to be effective in reducing anxiety severity,^22^ and another dissonance-based eating disorder prevention programme was successful in reducing depressive symptoms and negative affect.^23^ Some eating disorder prevention programmes did not have any effect on negative emotions.^24–26^

## Current study

Previous studies have mainly examined the effectiveness of eating disorder prevention programmes in reducing emotion-related risk factors, such as body dissatisfaction and dieting,^20,27–29^ or the co-occurrence of obesity and eating disorders.^30,31^ Only one systematic review has explored the effects of eating disorder prevention programmes on eating behaviours and depressive symptom across all age groups.^32^ However, there has not been a systematic review that specifically looks at the benefits of incorporating a mental health component into school-based eating disorder prevention programmes for adolescent mental health. Therefore, this review aims to summarise existing evidence on the impact of school-based eating disorder prevention programmes on adolescent mental health. The findings will be helpful in creating interventions to prevent maladaptive eating behaviours and negative emotions that often accompany such behaviours.

## Method

### Data sources

This systematic review followed the guidelines of the Preferred Reporting Items for Systematic Reviews and Meta-Analyses (PRISMA) and was registered with the Open Science Framework (https://doi.org/10.17605/OSF.IO/3QS2K). We searched databases (PubMed, PsycINFO, EMBASE and Web of Science) on 15 October 2023 for articles written in English since database inception, using the following search terms: (eating disorder OR anorexia nervosa OR bulimia nervosa OR binge-eating disorder) AND (school) AND (children OR adolescents) AND (intervention OR program) AND (mental health OR emotional outcomes).

### Study selection

Two authors (B.N.K.C. and S.I.L.) independently screened titles, then abstracts, before conducting a full-text review of potentially eligible studies. In instances where disagreements emerged, reviewers consulted each other and discrepancies were resolved by consensus. Articles were included if (a) the study evaluated the effect of an eating disorder prevention programme implemented in schools or universities on the mental health of adolescents using a randomised controlled trial (RCT), cluster RCT or quasi-experimental study design; and (b) the participants were aged 10–19 years with no self-reported or medical history of eating disorder. We excluded articles if (a) the programme targeted teachers or parents; or (b) results were reported in conferences, abstracts, editorials and/or letters only. The reference lists of previous systematic reviews and meta-analysis were also searched for relevant manuscripts.

### Data extraction and classification

Study characteristics and outcome measures were extracted on 18 October 2023. Two authors independently extracted the following data: (a) bibliographical data (author, publication year); (b) study characteristics (study period, end of study year, location, ethnicity, study type); (c) participants characteristics (study population characteristics, age, gender, sample size); (d) intervention design (name of intervention, content of intervention, availability of licensed professionals) and (e) emotional outcome measures.

### Quality assessment

Two authors (B.N.K.C. and S.I.L.) independently evaluated the quality of the included studies. The quality assessment adopted the checklist developed by the National Institute for Health and Care Excellence (NICE) for appraisal of quantitative interventions studies,^33^ with the following four criteria: (a) population (three items), (b) method of allocation to intervention (or comparison) (eight items), (c) outcomes (four items) and (d) analyses (five items). Items were rated on a scale of 0–2, with 0–13 points indicating poor quality, 14–27 points indicating fair quality and 28–40 points indicating high quality. The level of agreement between the two assessors was 98%, with any disagreements being resolved through discussion with the senior author (R.S.W.).

## Results

### Study selection

**Fig. 1:**
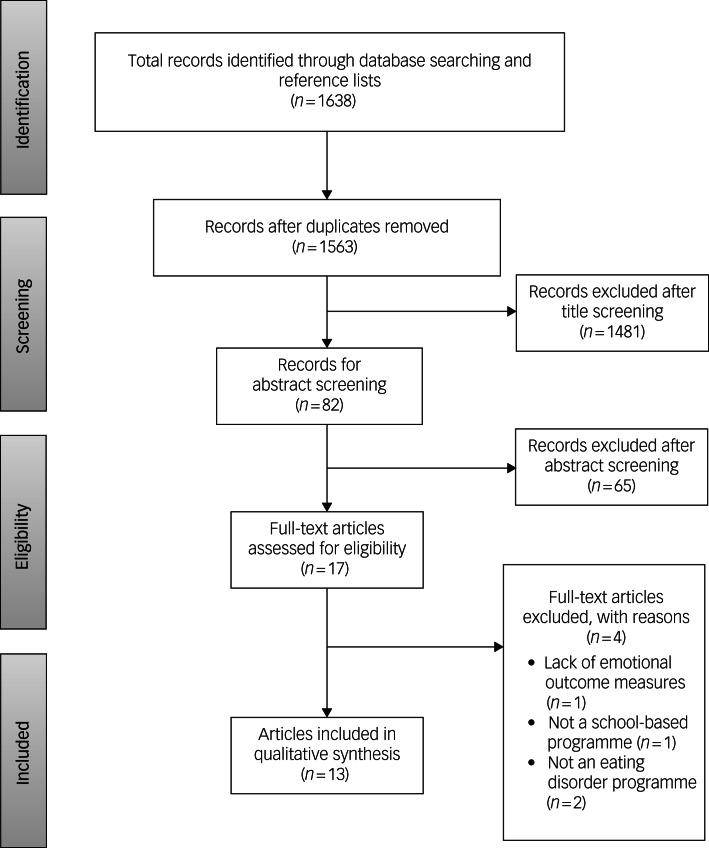
Study flowchart.

Figure 1 shows the results of the selection process. Databases were searched from inception until 15 October 2023. The searches yielded a total of 1638 articles, of which 75 were duplicates. The remaining 1563 titles and abstracts were assessed for eligibility. Of these, 17 articles were considered for full-text assessment, and 13 were included in the systematic review.^22–26,34–41^ Four articles were excluded because one had no emotional outcome measures, one was not a school-based programme and two were not an eating disorder programme. Since one article reported findings of two studies,^35^ the final number of studies included in this review was 14.

### Overall study characteristics

Table 1 shows the characteristics of the 14 included studies. They were all published between 1998 and 2020. The eating disorder prevention programmes were all conducted in Western countries. Five interventions took place in the USA,^34,35,39,41^ five in Australia,^24–26,36,37^ one in Germany,^22^ one in Switzerland,^38^ one in Brazil^23^ and one in Spain.^40^ The studies included in this review had sample sizes varying from 62 to 2001 participants, with ten interventions focusing on middle or high school students. Seven studies had a mixed-gender sample. Among the included studies, 12 were RCTs, whereas the remaining two utilised a quasi-experimental design. The studies reported a total of 11 mental health outcomes, including depressive symptoms, negative affect, perceived pressure, self-esteem and anxiety. Out of the 14 studies, eight provided a clear timeframe for follow-up. Only one study conducted a follow up study beyond 12 months, suggesting the need for longer follow-up periods in future research to evaluate the long-term sustainability of programme effects.

**Table 1:** Study characteristics

Author(s), year; country	Study design	Mean age, years (s.d.)	Gender	Conditions and sample size	Content of interventions	Total number of sessions	Session duration (mins)	Licensed professionals	Follow-up period (months)	Main results	Quality assessment score
Amaral et al (2019) Brazil^23^	RCT	16.25 (1.4)	Female	*n* = 141 1: DBI (*n* = 79) 2: Control (*n* = 62)	Body acceptance Stress coping skills	4	60	No	None	Depressive symptoms: post-test: *d* = 0.80 (*P* = 0.006) Negative affect: post-test: *d* = 0.75 (*P* = 0.013)	20
Atkinson and Wade (2015) Australia^36^	Cluster RCT	15.74 (0.82)	Female	*n* = 347 1: MBI (*n* = 138) 2: DBI (*n* = 108) 3: Control (*n* = 101)	DBI: Body acceptance Self-esteem enhancement Stress coping skills MBI: Body acceptance Mindfulness practice Stress coping skills	3	Not reported	No	1 and 6	The trained facilitator subset Perceived pressure: 6-month follow-up: DBI: *d* = 0.59 (*P* < 0.05) MBI: *d* = 0.47 (*P* < 0.05) Negative affect: not significant By risk status Perceived pressure: not significant Negative affect: DBI (low-risk group) post-test: *d* = 0.31 (*P* = 0.05)	29
Buddeberg-Fischer et al (1998) Switzerland^38^	Cluster RCT	16.1 (1.8)	Male and female	*n* = 314 1: Psychoeducation (*n* = 159) 2: Control (*n* = 155)	Body acceptance Healthy weight control skills Psychoeducation Sociocultural resistance skills/media literacy	3	90	Not reported	None	Overall distress: Female: *d* = 0.37 (*P* = 0.003) Psychological impairment: not significant	20
Gumz et al (2017) Germany^22^	Cluster RCT	Intervention group: 14.4 (0.30) Control group: 14.8 (0.35)	Male and female	*n* = 2001 1: Combined dissonance and mindfulness-based intervention (*n* = 1020) 2: Control (*n* = 981)	Body acceptance Healthy weight control skills Mindfulness practice Psychoeducation Self-esteem enhancement Social support Sociocultural resistance skills/media literacy Stress coping skills	3	90	Yes	6	Perceived pressure: post-test: *d* = 0.04 (*P* < 0.01) Anxiety: post-test: *d* = 0.11 (*P* < 0.02) Depressive symptoms: not significant	33
Johnson et al (2016) Australia^24^	Cluster RCT	13.63 (0.43)	Male and female	*n* = 308 1: MBI (*n* = 132) 2: Control (*n* = 176)	Mindfulness practice Stress coping skills	8	35–60	Yes	3	Anxiety: Male: 3-month follow-up: control < MBI: *d* = 0.22 (*P* < 0.01) Depressive symptoms: not significant Emotional dysregulation: not significant Well-being: not significant	30
Rohde et al (2014) USA^35^	RCT	12.5 (0.8)	Female	Programme 1: *n* = 81 1: DBI (*n* = 41) 2: Control (*n* = 40) Programme 2: *n* = 52 1: DBI (*n* = 27) 2: Control (*n* = 25)	Programmes 1 and 2: Body acceptance Healthy weight control skills Self-esteem enhancement	6	45	No	Programme 1: none Programme 2: 3	Negative affect: Programme 2: post-test: *d* = 0.60 (*P* = 0.04) Perceived pressure: Programme 1: post-test: *d* = 0.80 (*P* = 0.01)	Programme 1: 25 Programme 2: 31
Sepulveda et al (2007) Spain^40^	Quasi-experimental study	18.2 (0.5)	Male and female	*n* = 71 1: Healthy eating workshops (high risk) (*n* = 11) 2: Healthy eating workshops (low risk) (*n* = 23) 3: Comparison group (low risk) (*n* = 37)	Body acceptance Healthy weight control skills Self-esteem enhancement Sociocultural resistance skills/media literacy Stress coping skills	16	90	Yes	None	Mental pathology: not significant Self-esteem: not significant	12
Stice and Ragan (2002) USA^39^	Quasi-experimental study	Modal age: 21	Female	*n* = 66 1: Psychoeducation (*n* = 17) 2: Matched comparison group (*n* = 49)	Healthy weight control skills Psychoeducation Sociocultural resistance skills/media literacy	30	90	Not reported	None	Depressive symptoms: not significant	19
Stice et al (2013) USA^41^	RCT	21.6 (5.64)	Female	*n* = 408 1: DBI (*n* = 203) 2: Psychoeducational (brochure) control (*n* = 205)	Body acceptance Sociocultural resistance skills/media literacy	4	60	No	12	Negative affect: post-test: *d* = −0.53 (*P* < 0.001) 12-month follow-up: *d* = −0.38 (*P* < 0.001)	29
Wilksch and Wade (2009) Australia^25^	RCT	13.62 (0.37)	Male and female	*n* = 540 1: Media Smart (*n* = 233) 2: Control (*n* = 307)	Body acceptance Self-esteem enhancement Sociocultural resistance skills/media literacy	8	50	No	6 and 30	Self-esteem: Male: post-test: *d* = 0.25 (*P* < 0.05) Depressive symptoms: not significant Perceived pressure: not significant	24
Wilksch and Wade (2013) Australia^26^	Cluster RCT	12.71 (4.1)	Male and female	*n* = 114 1: Life Smart (*n* = 50) 2: Control (*n* = 64)	Healthy weight control skills Self-esteem enhancement Social support Stress coping skills	8	50	Yes	None	Depressive symptoms: not significant	19
Wilksch et al (2015) Australia^37^	Cluster RCT	13.21 (0.68)	Male and female	*n* = 1316 1: HELPP (*n* = 170) 2: Life Smart (*n* = 279) 3: Media Smart (*n* = 219) 4: Control (*n* = 473)	HELPP: Body acceptance Healthy weight control skills Sociocultural resistance skills/media literacy Life Smart: Healthy weight control skills Self-esteem enhancement Social support Sociocultural resistance skills/media literacy Stress coping skills Media Smart: Body acceptance Sociocultural resistance skills/media literacy Stress coping skill	8	50	No	6 and 12	Depressive symptoms: Male: 6-month follow-up: Media Smart < Life Smart: *d* = 0.51 (*P* < 0.05) HELPP < life smart: *d* = 0.46 (*P* < 0.05) 12-month follow-up: Media Smart < control: *d* = 0.39 (*P* < 0.05) Perceived pressure: Female: 6-month follow-up: Media Smart < HELPP: *d* = 0.45 (*P* < 0.05) Control < HELPP: *d* = 0.44 (*P* < 0.05)	23
Wilson et al (2020) USA^34^	RCT	20.6 (2.9)	Female	*n* = 94 1: DBI (*n* = 41) 2: Psychoeducational (brochure) control (*n* = 53)	Body acceptance Healthy weight control skills	2	90–120	No	1	Mental health-related quality of life: not significant	27

RCT, randomised controlled trial; DBI, dissonance-based intervention; MBI, mindfulness-based intervention; HELPP, Helping, Encouraging, Listening and Protecting Peers.

### Study quality

According to the predefined scoring system based on the quality assessment checklist developed by NICE,^33^ we classified five studies as high quality,^22,24,35,36,41^ eight studies as fair quality^23,25,26,34,35,37–39^ and one study as poor quality.^40^ Most of the studies were well defined and provided a clear description of population, areas and interventions. However, five studies did not have a representative population. All studies randomly allocated or matched participants, but only three studies reported that their assessors were blind to intervention allocation. Although all studies used reliable outcome measures, nine studies did not give details about sample size estimation.

### Design of the interventions

Three studies^35–37^ covered more than one intervention, whereas the remaining studies focused on a single intervention, resulting in a total of 17 interventions. Among these 17 eating disorder prevention programmes, ten programmes demonstrated significant improvement in mental health outcomes, and seven programmes did not yield significant results. These 17 programmes can be categorised into seven types, which are dissonance-based interventions, mindfulness-based interventions, combined dissonance and mindfulness-based interventions, Media Smart, Life Smart, the HELPP (Helping, Encouraging, Listening and Protecting Peers) initiative, and eating disorder classes or workshops in high school/university. The topics covered in these class/workshops include body acceptance, healthy eating, nutrition, exercise, self-esteem enhancement, sociocultural resistance skills, media literacy, stress coping skills, psychoeducation, social support and mindfulness practice. Regarding dissonance-based interventions, the goal is to create cognitive dissonance by having participants express and behave in ways that oppose the thin-ideal standard of female beauty. This is done through group activities and homework assignments. On the other hand, Life Smart promotes the idea that health goes beyond just weight, eating and exercise. It covers topics like physical activity, sleep, cognitive styles, emotional management and social support, targeting risk factors for weight gain beyond traditional targets. Media Smart focuses on addressing the risk factor of media internalisation, where individuals rigidly adhere to societal ideals of size and appearance. HELPP targets the risk factors of internalising appearance ideals and engaging in appearance comparisons. All three programmes are developed based on evidence-based principles, including interactivity, avoiding solely providing psychoeducation about eating disorders and obesity, and featuring multiple sessions with eight lessons of 50 min each, delivered at a pace of two lessons per week. Although Media Smart and HELPP target similar eating disorder risk factors, Life Smart addresses a wider range of shared risk factors as well as obesity-related risk factors.

### Characteristics of studies reporting significant intervention effects

Ten intervention programmes were found to be effective in reducing depressive symptoms, negative affect, perceived pressure, anxiety and distress among adolescents. These included five dissonance-based interventions,^23,35,36,41^ two Media Smart programmes,^25,37^ one mindfulness-based intervention,^36^ one programme with a combined dissonance and mindfulness-based approach,^22^ and one psychoeducational intervention in high school.^38^

### Intervention duration

The effective programmes had a shorter intervention duration compared with the programmes that were reported to have no effects. Dissonance-based interventions had around three to six sessions, with each session lasting approximately 45–60 min. The combined dissonance and mindfulness-based intervention and the psychoeducational intervention in high school consisted of three sessions (90 min per session). The two Media Smart interventions had eight 50 min sessions. One dissonance-based and one mindfulness-based intervention programme had three sessions, but the duration of each session was not provided.

### Licensed professionals

Of the ten effective programmes, eight programmes were administered by non-licensed facilitators supervised by healthcare professionals. The psychoeducational intervention in high school did not provide any details about the facilitator's training background. The combined dissonance and mindfulness-based programme was the only effective programme that was delivered by licensed professionals, such as members of the Center of the Prevention of Addiction or research team members who held at least a Master's degree in clinical psychology.

### Intervention content

All of the effective programmes addressed the concept of body acceptance and consistently resulted in a reduction of negative emotional outcomes among adolescents following the intervention. These programmes also covered topics such as healthy eating, nutrition, exercise, self-esteem, sociocultural resistance skills, media literacy, stress coping strategies, social support, psychoeducation and mindfulness practice. The intervention content was delivered through a range of methods, including games, videos, role plays and discussion.

### Patient characteristics

Table 1 shows the age and gender of the participants in each effective programme. The combined dissonance and mindfulness-based intervention recruited both female and male adolescents, and resulted in a significant decrease in anxiety for both genders after the intervention. However, in the two Media Smart programmes, only male participants showed improvement in self-esteem and depressive symptoms following the intervention. Among the five dissonance-based interventions, negative affect was reduced in female adolescents. The mindfulness-based intervention, delivered by trained facilitators, was also effective in female adolescents. Similarly, the psychoeducational intervention in high school yielded similar positive results in female participants.

### Characteristics of studies reporting no intervention effects

Seven intervention programmes were found to have no significant impact on adolescent mental health outcomes, including anxiety and depressive symptoms. These included two Life Smart programmes,^26,37^ two eating disorder classes/workshops in university,^39,40^ one HELPP programme,^37^ one mindfulness-based intervention^24^ and one dissonance-based intervention.^34^

### Intervention duration

These ineffective programmes varied substantially with respect to intervention and session duration. The dissonance-based intervention only had two sessions, with each session lasting approximately 90–120 min. The mindfulness-based intervention programme had eight 35–60 min sessions. The Life Smart and HELPP programmes consisted of eight sessions, with each session lasting 50 min. The two eating disorder classes/workshops in university involved 30 and 16 sessions (90 min per session), respectively.

### Licensed professionals

Of the seven ineffective programmes, three were delivered by licensed professionals, such as mindfulness practitioner, clinical psychologists and child and adolescent psychiatrists. One programme did not provide any information regarding the training background of the facilitator.

### Intervention content

All of the effective programmes addressed the concept of body acceptance, whereas only three of the ineffective programmes included this topic. The ineffective programmes also covered other topics presented in the effective programmes.

### Patient characteristics

A female-only evaluation was conducted for one dissonance-based intervention and one eating disorder class in university, whereas the other five programmes were evaluated in both female and male adolescents.

## Discussion

This review examines the impact of school-based eating disorder prevention programmes on the mental health of adolescents, based on 14 studies. Among the seven different types of intervention identified, five were found to be effective in reducing negative emotions in adolescents: dissonance-based intervention, combined dissonance- and mindfulness-based programme, mindfulness-based intervention, Media Smart and eating disorder classes in high school.^22,23,25,35–38,41^ These intervention types had a relatively shorter intervention period and could be delivered by non-licensed professionals, compared with the other two types (Life Smart and HELPP). The findings suggest that promoting body acceptance can help alleviate negative emotions and self-perceptions. They also highlight the limited availability of interventions specifically targeting male adolescents.

Our findings indicate that the effectiveness of a school-based eating disorder prevention programme in improving adolescent mental health depends on several programme characteristics, including the number and duration of sessions, intervention content and involvement of licensed professionals. We found no evidence of a significant association between longer programme duration and greater reductions in negative emotions among adolescents. For example, the longest programme, which consisted of 30 sessions lasting 90 min each, did not have any impact on mental health. Conversely, the five effective intervention types typically had fewer sessions (around three to eight) and shorter session durations (45–90 min). A previous meta-analysis on dissonance-based eating disorder prevention programmes has indicated that the optimal number of sessions for positive mental health effects is four to five.^42^ Another systematic review also highlighted that significant improvements are usually observed in programmes that involve a total of 5 h of teaching.^43^ Therefore, a recommended intervention time would be around four to five lessons or a total of five activity hours, as this allows participants to reinforce their newly acquired knowledge on eating disorder prevention. This knowledge may include topics such as developing healthy eating habits, fostering body acceptance, building self-esteem and understanding the importance of seeking help and support when needed. By providing this intervention time, participants can better internalise the information and skills, leading to the promotion of social and emotional well-being.

Interestingly, we found that having licensed professionals involved in delivering an eating disorder prevention programme was not necessary for improving the mental health of adolescents. Among the five effective intervention types, only one (the combined dissonance- and mindfulness-based intervention) was led by licensed professionals. Previous studies have suggested that programmes led by clinicians may be more effective in reducing negative emotions compared with those led by researchers or peers.^42,44^ However, programmes led by licensed professionals can be costly and their availability may be inconsistent, which can affect the success of the programme. On the other hand, research has shown that peer-led dissonance-based eating disorder prevention programmes can also effectively reduce negative emotion.^44,45^ In fact, programmes led by trained non-licensed facilitators have been found to be more cost-effective in reducing negative emotions compared with programmes led by licensed professionals such as teachers and school counsellors.^20^ Therefore, future eating disorder prevention programmes could consider utilising non-licensed facilitators supervised by healthcare professionals to provide cost-effective services.

The reviewed school-based eating disorder prevention programmes covered various topics such as psychoeducation, body acceptance, sociocultural issues, nutrition and physical activities, self-esteem, stress coping, mindfulness practice and social support. Among the topics covered in the programmes that had a positive impact on the mental health of adolescents, only body acceptance was consistently addressed. This aligns with previous research indicating that programmes focusing on body acceptance are more effective in reducing negative affect.^20^ Adolescents are particularly vulnerable to societal norms, peer influence and social media,^46,47^ which can contribute to poor body image and low self-esteem.^48–53^ Therefore, addressing self-esteem has the potential to improve mental health outcomes. The dual pathway model suggests that body dissatisfaction can increase the risk of eating disorders through unhealthy dieting and negative affect.^23^ Dissonance-based approaches, which aim to modify false beliefs, are frequently used in eating disorder prevention programmes.^22,23,35,36,41^ These interventions challenge cultural beauty standards through verbal, written and behavioural exercises, creating cognitive dissonance. This helps to reduce negative emotions and body dissatisfaction.^54^ However, it may also cause anxiety and discomfort in some individuals. Therefore, it is important to have trained professionals design and implement eating disorder prevention programmes to offer support and guidance to participants throughout the process. Notably, schools offer an ideal platform for policy makers and educators to educate young individuals on body acceptance and inform them about the harmful effects of having a negative body image, which can lead to developing eating disorders. After receiving training, these young individuals can then share their knowledge with their peers in the future.

Another noteworthy finding is the limited availability of eating disorder prevention programmes specifically designed for male adolescents. Recent research has shown an increase in disordered eating concerns among male adolescents because of their desire for muscularity and societal body ideals.^55,56^ Future eating disorder prevention programmes should incorporate more content addressing the risk factors related to muscularity, such as teaching male adolescents to accept their bodies and cope with the pressures of masculinity. The Media Smart programme, for example, utilised these approaches and showed positive effects on self-esteem and depression outcomes among male adolescents.^25,37^

This review had several strengths, such as analysing various types of school-based eating disorder prevention interventions and distinguishing effective and ineffective programmes for enhancing the mental health of adolescents. Moreover, it included a large number of participants (*n* = 5853) and identified the key features and topics of effective interventions. These findings will help in designing future eating disorder prevention programmes.

However, this review also had limitations. First, because of the small number of studies available and significant variability among them, we were unable to effectively combine and analyse the results using quantitative methods. Second, the majority of the included interventions lacked information on the blinding of participants or investigators. Additionally, there was no ascertainment of the eating disorder diagnosis in participants, making it difficult to accurately estimate biases in the findings because of insufficient details. Third, some interventions did not specify the follow-up time, preventing us from drawing conclusions on the long-term effects. Finally, most of the interventions analysed in this review were carried out in Western countries. It is worth mentioning that cultural standards of beauty can differ between Western and non-Western populations. Therefore, the results obtained from Western populations may not be applicable to non-Western populations. Conducting additional studies on non-Western populations can assist in identifying particular risk factors that are pertinent to them and enhancing the effectiveness of eating disorder prevention programmes for non-Western participants.

In conclusion, negative emotions and mental health problems are recognised as risk factors for eating disorders. School-based initiatives targeting eating disorder prevention in teenagers should include a mental health component that educates students about body acceptance in four to five lessons. To enhance cost-effectiveness and sustainability, these programmes can be implemented by trained facilitators who are supervised by healthcare professionals. In addition, it is important for eating disorder prevention programmes to target both male and female adolescents, helping them recognise and change their distorted beliefs about body shape. This can lead to better mental health outcomes.

## Data Availability

The data that support the findings of this study are available on request from the corresponding author, R.S.W.
